# Psychometric Evaluation of the Japanese 9-Item Shared Decision-Making Questionnaire and Its Association with Decision Conflict and Patient Factors in Japanese Primary Care

**DOI:** 10.31662/jmaj.2019-0069

**Published:** 2020-07-07

**Authors:** Yuko Goto, Hisayuki Miura, Daisuke Son, Hidenori Arai, Levente Kriston, Isabelle Scholl, Martin Härter, Kotaro Sato, Tesshu Kusaba

**Affiliations:** 1 Department of Home Care and Regional Liaison Promotion, National Center for Geriatrics and Gerontology of Japan, Aichi, Japan; 2 Department of Medical Education Studies, International Research Center for Medical Education, Graduate School of Medicine, The University of Tokyo, Tokyo, Japan; 3 National Center for Geriatrics and Gerontology of Japan, Aichi, Japan; 4 Department of Medical Psychology, University Medical Center Hamburg-Eppendorf, Hamburg, Germany; 5 The Hokkaido Center for family medicine, Hokkaido, Japan

**Keywords:** communication, primary health care, questionnaire, shared decision-making

## Abstract

**Introduction:**

This study aimed to verify the internal consistency and validity of the Japanese version of the 9-item Shared Decision-Making Questionnaire (SDM-Q-9) and investigate the association among patient factors, shared decision-making experienced by patients, and patients’ decision conflict during the treatment decision process in primary outpatient settings in Japan.

**Methods:**

Patients who visited a primary care outpatient unit for the first time and completed the Japanese version of SDM-Q-9 and the Decisional Conflict Scale (DCS) immediately after consultation were included. The internal consistency of SDM-Q-9 was assessed using Cronbach’s alpha coefficient. Factor analysis and structural equation modeling were used to investigate structural construct validity. The relationship among patient-perceived experiences of shared decision-making, decision conflict, and patient factors was evaluated using correlation analysis.

**Results:**

A total of 131 patients with chronic diseases (55.0% females, 28.2% aged ≥ 70 years) were included in this analysis. Cronbach’s alpha for the Japanese version of SDM-Q-9 was 0.917, indicating a high degree of internal consistency. Confirmatory factor analysis indicated that the Japanese version of SDM-Q-9 had a one-factor structure. Spearman’s rank correlation analysis indicated that the correlation between SDM-Q-9 and DCS was −0.577 (*p* < 0.05), indicating a significant inverse correlation and convergent validity. Older age was positively associated with perceived support of the physician in understanding all information.

**Conclusions:**

We confirmed that the Japanese version of SDM-Q-9 was both reliable and valid for use in Japanese primary care settings. In addition, we found a clear association between shared decision-making and decisional conflict of patients.

## Introduction

In developed nations such as Germany, the United States, and the Netherlands ^(1)^, communication education for professionals is included in the training, national medical policies, and guidelines to facilitate shared decision-making (SDM) regarding the treatment and care as measured by the patients’ perspective ^(2)^.

There are three main approaches to decision-making through communication: (1) paternalistic decision-making, in which the medical professional makes decisions; (2) “informed decision-making,” in which the patient makes decision after receiving information from the medical professional; and (3) SDM, in which the medical professional and patient make decisions together. Recently, there has been a shift from informed decision-making to SDM, wherein medical professionals try to understand the patient’s values and preferences in a better way and involve the patient in making medical and care choices ^(3)^.

To date, paternalistic decision-making has been widely used in Japan ^(4)^. In addition, patients and family members prefer to leave treatment and nursing care decisions solely on medical professionals, which acts as a barrier for the practical application of SDM. However, at present, there is a high degree of patient’s self-awareness in Japan regarding the “right to freedom of choice” and “right to self-determination,” as defined in the World Medical Association Declaration of Lisbon on the Rights of the Patient ^(5)^. Consequently, there is a growing need to provide medical treatment and nursing care that cater to patient’s values and preferences.

Thus, SDM research designed to develop new approaches of decision-making support is needed. Given that medical professionals often experience cases wherein they cannot decide the treatment without understanding the patient’s values and preferences, they typically find themselves in a dilemma while providing patient-tailored medical treatment and nursing care. To address this dilemma, there is a need to define SDM operationally and initiate a quantitative study SDM.

In developed nations, several scales have been developed for measuring SDM ^(6), (7)^. The 9-item Shared Decision-Making Questionnaire (SDM-Q-9) is one of the scales for measuring SDM; it uses patient-reported experience measure (PREM) to measure concepts in nine stages. It has been translated into 27 languages ^(8)^ and is used in various specialties, including primary care, oncology, pediatrics, psychiatric illnesses, and preventive settings. Its validity has been confirmed in various language and cultural settings, and it is considered to be highly compatible with several cultures.

Most previous studies on decision-making have measured outcomes such as decisional conflict, decision-related satisfaction, decision-related regret, participation in decision-making, and quality of life ^(6), (9)^. These studies have confirmed that SDM leads to reduced decision-related regret and conflict, better understanding of the treatment, increased satisfaction and trust in the treatment, improved quality of life, reduced anxiety, and behavioral changes in patients, such as improved adherence to treatment ^(3), (9)^. However, these studies have been conducted outside Japan, and the benefits of SDM in Japan have not been examined. Therefore, the present study aimed to evaluate the Japanese version of the SDM-Q-9 and use it to explore the relationship among patient-perceived SDM experiences, decisional conflict, and patient factors during treatment-related decision-making in Japanese primary care settings.

## Materials and Methods

### SDM-Q-9

SDM-Q-9 is a questionnaire-based measurement of a 9-stage model ^(10)^ constructed using characteristics ^(11)^ that are considered important in SDM. The original version of SDM questionnaire was developed in 2010 at the University of Hamburg. MH, IS and LK of co-authors have developed the SDM-Q-9 in its original German version. The internal consistency and validity of the original and multiple translated versions of SDM-Q-9 have been verified ^(7)^. SDM is currently defined as a joint decision-making method that entails a sharing of the patient’s values and preferences between patient and medical professionals to decide upon one or more available choices.

### Translation of the questionnaire

The Japanese version was developed after obtaining approval from the developers of the original version. Two German-to-Japanese professional translators independently prepared preliminary translations. The decision-making researchers integrated the two translated into one, and seven individuals who were either decision-making researchers or patients’ association representatives used the Delphi method to verify the face validity of the contents. The patient’s association representatives were recruited, because it is crucial that the expressions in the questionnaire can be understood by the general patient. The Japanese version was then back-translated into German by a professional translator. The back-translation was checked by the members of the University of Hamburg who made original German version. The above procedure was repeated twice. Finally, the team that developed the original version granted its approval for the Japanese version. Responses to the 9 questions were scored using a 6-point Likert-type set of multiple choices ranging from “completely disagree” (0 points) to “completely agree” (5 points) for a total possible maximum score of 45 points. Higher scores indicated a higher degree of SDM.

### Japanese version of the decisional conflict to assess convergent validity

In addition to the Japanese version of SDM-Q-9, we used the Japanese version of the Decisional Conflict Scale (DCS), which measures the degree of conflict experienced by the patient when making treatment-related decisions ^(12)^. The original version of DCS was developed at the University of Ottawa, Canada. This scale comprises 16 questions, which are answered by respondents using a 5-point Likert scale and has 5 subscales: information sharing, clarifying values, supporting decisions, confidence in decisions, and satisfaction with decisions ^(13)^. The total score for DCS was calculated by adding the scores for all the 16 questions, followed by dividing the total scare by 16 and then multiplying by 25 ^(14)^. The scores ranged from 0, which indicated “no conflict,” to 100, which indicated “extremely high degree of conflict.”

The internal consistency and validity of both the original and Japanese version of DCS have been verified. In the Japanese version, the response “strongly agree” was assigned 0 points and “strongly disagree” 4 points. Higher scores indicate higher levels of decisional conflict. Scores of 37.5 or more indicate that the patient’s decision-making process requires support.

### Study design and setting

This study was conducted using a self-administered questionnaire survey at the Centre for Family Medicine Development and the Hokkaido Centre for Family Medicine (2 medical corporations, involving 23 outpatient clinics), which includes Japan Primary Care Association-certified family physician training program, given that many primary care specialists are trained in communication skills. Primary care settings provide medical care for routine illnesses and health problems. The questionnaire was administered to the patients on their first visit. In contrast to the non-routine medical care provided at emergency medicine and acute-care hospitals, primary care involves general medical care. In many cases, primary care involves decisions on medical treatment by sharing information about the patients, values, and lifestyle preferences.

### Study participants, recruitment, sample size, and study period

Patients aged ≥20 years who were undergoing their initial consultation at a primary care setting were recruited for this study. The inclusion criteria were as follows: (1) patients undergoing their initial consultation with a physician in charge of outpatient care whom they had never met before and (2) patients with a stable medical condition that was chronic and not life-threatening. The exclusion criterion was patients with illnesses and symptoms, considered to hamper communication. In Japan, patients are required to complete a consultation-related questionnaire prior to the physical examination at the first consultation. Thus, prior to performing physical examination, the staff of the medical facility and outpatient physician had an opportunity to describe this study to the patients. The attending physician asked only the patients who gave the oral informed consent in this prior explanation to complete the questionnaire after consultation.

In previous studies on the development of multi-language versions of SDM-Q-9, the target sample size was based on the heuristic approach in 15-20 patients per item of the questionnaire ^(7)^. In this study, we determined the sample size as 150 considering the feasibility. The study was conducted for 12 months from June 2016 to June 2017.

### Ethical considerations

This study was conducted in accordance with the guidelines of the Declaration of Helsinki and was conducted after receiving approval from the Institutional Review Board of the National Center for Geriatrics and Gerontology (no. 913). All questionnaires were anonymously completed and did not comprise any participant’s name or any other personal information. The patient handed the completed questionnaires to staff members of the clinic in sealed envelopes, and these staff members could not see the completed questionnaires.

### Statistical analysis

The Japanese version of SDM-Q-9 and DCS were scored and analyzed using descriptive statistical analysis.

The Japanese version of SDM-Q-9 was assessed for internal consistency by calculating the Cronbach’s alpha coefficient (α). In accordance with the procedure used in the original version, the raw score of the Japanese version of SDM-Q-9 was multiplied by 20/9 to convert it to a score out of a maximum possible score of 100. In care all SDM-Q-9 scores are true, 45 points are obtained in total, and it is difficult to quickly determine whether a missing value or the degree of SDM is low; however, by scoring 100 points, it is possible to determine this ^(10)^. We used this multiplied value for analysis. 

To account for the cultural differences between Japan and European countries, including Germany, we conducted exploratory factor analysis. Exploratory factor analysis was conducted by performing factor analysis using principal factor analysis. We calculated the correlations for the whole instrument. 

We performed confirmatory factor analysis using structural equation modeling. Local and global goodness-of-fit indices were calculated and assessed using established rules to estimate the model fit. We used Spearman’s rank correlation analysis to verify the relationship between the Japanese version of SDM-Q-9 and subordinate concepts of the Japanese version of DCS. Lower DCS scores indicated less decisional conflict, and higher SDM-Q-9 scores indicate higher degrees of SDM. DCS comprises five subscales: information sharing, clarifying values, supporting decisions, confidence in decisions, and satisfaction with decisions. We tested whether each DCS subscale score correlated with the total SDM score. 

The relationship between patient characteristics and SDM-Q-9 was analyzed using the Spearman’s rank correlation test and multivariate regression analysis. Patient characteristics included patient age, sex, education, marital status, and employment status. Spearman’s rank correlation test was used to examine the relationship between patient age groups (≥70 versus ≤69 years), sex (male versus female), education (high school, university or above, junior high school, college of technology or no answer), current marital status (with or without a legal spouse), employment status (worked, educated, housewife, after retirement or others), and the nine SDM questions.

All analyses were performed using IBM SPSS Statistics version 25 and IBM SPSS Amos Graphics version 25 software programs (Chicago, IL, USA). A P value of <0.05 was considered statistically significant.

## Results

### Study sample

We received questionnaire responses from 142 patients who visited the 23 outpatient clinics for initial consultation and who provided consent for participation. We then analyzed data from 131 individuals with no missing data in either SDM-Q-9 or DCS.

The majority of the patients (58.7%) were aged 50-70 years. The proportion of women was slightly higher than that of men, and more than 70% of the patients had completed either high school or university level education (Table 1).

**Table 1.** Characteristics of Patients Included in the Study (N = 131).

Feature	Category	Number (%)
Age (year)	20s-60s	93 (70.8)
	≥70s	37 (28.2)
	No answer	1 (1.0)
Sex	Female	72 (55.0)
	Male	58 (44.3)
	No answer	1 (0.7)
Education	High school	48 (36.6)
	University or above	47 (35.9)
	Junior high school	19 (14.5)
	College of technology	16 (12.2)
	No answer	1 (0.8)
Marital status	Married	79 (60.3)
	Unmarried	52 (39.7)
Employment status	Employed	65 (49.6)
	Housewife	33 (25.2)
	After retirement	19 (14.5)
	Other	13 (9.9)
	No answer	1 (0.7)

### Internal consistency analysis of the Japanese version of the SDM-Q-9

Cronbach’s alpha of the Japanese version of the SDM-Q-9 was 0.917. All corrected item-total correlations exceeded 0.89 (Table 2).

**Table 2.** Item Characteristics of the Japanese Version of SDM-Q-9 (N = 131).

	Minimum value	Maximum value	Mean (median)	SD	Cronbach’s α if item deleted	I-T correlation	Factor loading
SDM1．My doctor made clear that a decision needs to be made.	0	11.11	9.79 (11.11)	1.945	0.901	0.755	0.618
SDM2．My doctor wanted to know exactly how I want to be involved in making the decision.	0	11.11	10.08 (11.11)	1.771	0.908	0.649	0.449
SDM3．My doctor told me that there are different options for treating my medical condition.	0	11.11	9.52 (11.11)	2.343	0.898	0.794	0.747
SDM4. My doctor precisely explained the advantages and disadvantages of the treatment options.	0	11.11	9.19 (11.11)	2.655	0.897	0.816	0.780
SDM5. My doctor helped me understand all the information.	6.667	11.11	10.14 (11.11)	1.325	0.914	0.571	0.312
SDM6．My doctor asked me which treatment option I prefer.	0	11.11	9.94 (11.11)	1.936	0.907	0.670	0.471
SDM7．My doctor and I thoroughly weighed the different treatment options.	0	11.11	8.77 (8.89)	2.774	0.902	0.765	0.658
SDM8．My doctor and I selected a treatment option together.	0	11.11	9.75 (11.11)	1.979	0.901	0.756	0.569
SDM9．My doctor and I reached an agreement on how to proceed.	0	11.11	10.26 (11.11)	1.594	0.910	0.619	0.368

### Factorial validity analysis of the Japanese version of SDM-Q-9

The results of exploratory factor analysis using principal factor analysis suggested that the Japanese version of SDM-Q-9 comprises a one-factor structure, similar to the original version. Factor loadings exceeded a score of 0.4 for seven of the nine items.

First, analysis with a model assuming the absence of residual correlation for the one-factor structure revealed a chi-squared value (discrepancy chi-square value divided by the degrees of freedom) of χ^2^ = 182.357 (*p *= 0.001), goodness-of-fit index (GFII) = 0.771, adjusted goodness-of-fit index (AGFI) = 0.619, root mean square error of approximation (RMSEA) = 0.210, and comparative fit index (CFI) = 0.809. These findings indicated that the model did not fit the actual data very well. Therefore, the model was modified to assume the presence of residual correlation based on the goodness-of-fit of the model and clinical viewpoint.

**Figure 1.**
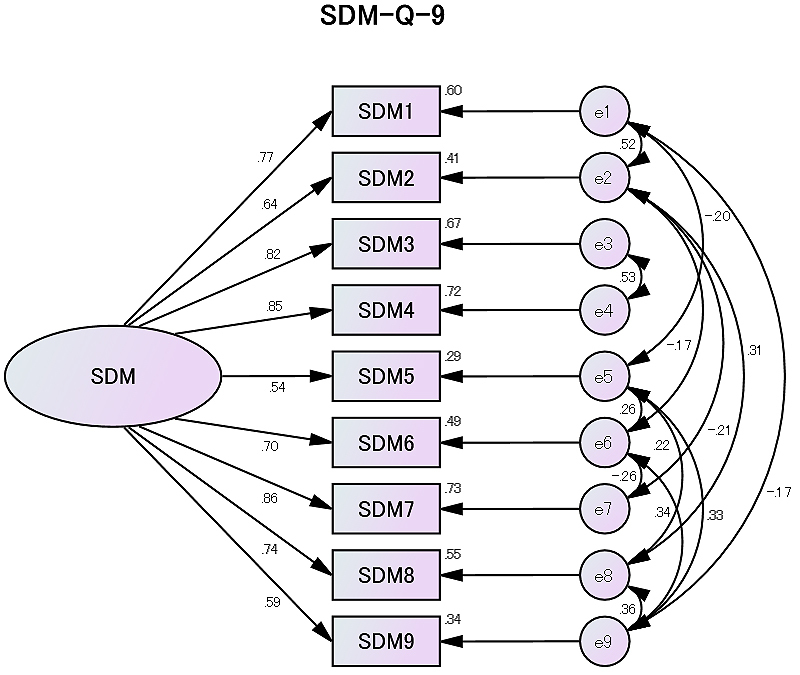
The confirmatory factor model of the Japanese version of the 9-item Shared Decision-Making Questionnaire (SDM-Q-9). Values on the single-headed arrows are partial standardized regression weights. Values on the double-headed arrows are correlation coefficients. A higher value indicates a stronger causal relationship. The circles of e1-e9 indicate errors (measurement residual).

Next, confirmatory factor analysis resulted in a chi-squared value (discrepancy chi-square value divided by the degrees of freedom) of 11.84 (*p *= 0.619), GFI of 0.981, AGFI of 0.938, RMSEA of 0.0, and CFI of 1.0, suggesting good fit to the data (Figure 1).

### Relationship between SDM-Q-9 and DCS (Convergent Validity)

The distribution of DCS is presented in Table 3. Spearman’s rank correlation analysis revealed that the correlation coefficient between the total SDM score and total DCS score was −0.577 (*p* < 0.05), indicating a strong inverse correlation between the two scores.

**Table 3.** The Distribution of the Decisional Conflict Scale (DCS) Subscores.

	DCS1	DCS2	DCS3	DCS4	DCS5	DCS6	DCS7	DCS8	DCS9	DCS10	DCS11	DCS12	DCS13	DCS14	DCS15	DCS16
Mean	0.89	0.99	1.19	1.02	1.31	1.2	1.47	0.75	0.88	0.96	1.24	1.13	0.93	0.95	1.18	0.95
Median	1	1	1	1	1	1	1	1	1	1	1	1	1	1	1	1
SD	0.767	0.78	0.833	0.818	0.902	0.836	1.076	0.768	0.841	0.854	0.977	0.915	0.834	0.821	0.811	0.797
Minimum value	0	0	0	0	0	0	0	0	0	0	0	0	0	0	0	0
Maximum value	3	4	4	4	4	4	4	3	4	4	4	4	4	4	3	3

The correlation coefficient values between the total SDM score and five DCS subscale scores were as follows: information sharing, −0.508 (*p* < 0.05); clarifying values, −0.646 (*p* < 0.05); supporting decisions, −0.426 (*p* < 0.05); confidence in decisions, −0.393 (*p* < 0.05); and satisfaction with decisions, −0.514 (*p* < 0.05).

### Relationship between SDM and patient characteristics

Spearman’s rank correlation analysis revealed that only SDM question 5, i.e., “My doctor helped me understand all the information” (supporting comprehension of the received information), was correlated with patient age (r = 0.263, *p *= 0.003), marital status (r = 0.211, *p *= 0.015), and employment status (r = 0.201, *p *= 0.022). To confirm the observed correlations of patient age, marital status, and employment status with SDM question 5, we performed forced-entry multiple regression analysis on these patient factors and found that the multiple determination coefficient was 0.083 (p < 0.05), the standardization coefficient for patients was β = 0.217 (p = 0.033), and the standardization coefficient for marital status was β = 0.187 (p = 0.032) (Table 4). According to these results, older patients and married patients perceived a higher level of support from their physician about understanding the information they received.

**Table 4.** Results of Multiple Regression Analysis Conducted Using Patient Factors and SDM Question 5.

Variables	B	SE B	β	*p* values
Patient age (≥70 years)	0.642	0.298	0.217	0.033
Marital status (married)	0.503	0.231	0.186	0.032
Employment status (employed)	0.120	0.270	0.045	0.658

B, nonstandardized coefficient; SE B, Standard deviation of nonstandardized coefficient; β, standardized coefficient

Statistical analysis indicated that the variance inflation factor of patient age and marital status was 1.044.

## Discussion

### Factorial structure of the Japanese version of SDM-Q-9

Based on the results of this study, we found that the Japanese version of SDM-Q-9 had a one-factor structure, which was similar to that of the original version (German version) and English version developed by the same team. In addition, the results showed that the eight-question versions (Spanish ^(15)^, Dutch ^(16)^, Arabic ^(17)^, and Cantonese ^(18)^ versions) were better suited than the nine-question versions. However, a previous study ^(19)^ has used the nine-question version of the Spanish SDM, and both eight- and nine-question versions may be available in this language.

Medical communication education is being conducted in various settings, most notably in the developed nations worldwide; thus, it is possible that the health literacy of patients regarding communication with medical practitioners varies ^(20)^. Slight semantic variations in the SDM scale have also been suggested as it is available in many languages and is used in many cultures ^(21)^. Through investigations in various educational and medical settings, the Japanese version should undergo validation for conceptual configuration and linguistic refinement as needed.

### Need for measuring decisional conflict and shared decision-making

In this study, the correlation coefficient between the total SDM score and total DCS score indicated a strong inverse correlation between the two scores. To the best of our knowledge, this is the first study to examine SDM and decisional conflict in Japanese primary care settings. Decisional conflict was measured using patient-reported outcome measure, wherein which patients reported how they felt about the decision made. SDM was evaluated using PREM, wherein which patients reported their experience on the process of decision-making. This time, a clear correlation was confirmed between the total SDM score and all the five subscales of DCS. Therefore, the influence of the outcomes of decision (conflict of decision) and decision process (SDM) reported by patients was confirmed, and phenomena similar to other cultural and linguistic spheres were also identified in Japan. The outcomes and experience information obtained from the patients’ viewpoint have been found to be closely associated with those reported by other studies on patient-centered care ^(22), (23)^. In Japan, efforts to assess the quality of medical treatment and patient care by assessing patient experiences are not very common ^(24)^. However, it is important to carefully collect information that reflects patients’ viewpoints and utilize this information to improve medical treatment and patient care. Anxieties, conflicts, and decision-making processes, which are evaluated based on patient reports, can be susceptible to being influenced by the communication skills of medical professionals. Thus, there is a need for effective communication between patients and medical professionals to comprehensively assess and understand the patient’s viewpoints ^(25)^.

### Patient factors associated with SDM

In this study, we found that patient age (≥70 years) and marital status (married) were significantly associated with SDM question 5: “My doctor helped me understand all the information.” 

Another study has reported that Japanese patients desire additional explanations on specialized medical terms and foreign-origin terms ^(26)^. In the present study, older patients might acknowledge the physician’s help in clarifying complex terms to a greater extent than other patients, and they might have a strong awareness of SDM question 5. Other subquestions did not differ by age. Participating physicians are trained in decision-making support via specialist education courses and may be proficient in sharing decision-making and understanding patient’s values regardless of age. In Japan, which is experiencing a continuous increase in the number of older patients, physicians can be required to possess SDM skills to support such patients by helping them understand complex medical information.

In the present study, we found no differences between male and female patients. Rather than patient sex, marital status can more likely affect SDM in medical settings. Previous studies have not revealed the relationship between marriage and SDM. In Japan, a patient’s spouse often makes patient’s treatment decision and was inserted to explore the implications of family influences and treatment decisions. A characteristic feature regarding the marital status in Japan is that a larger number of older people are married compared with younger ones. Thus, although age and marital status may be influential factors, this issue requires further investigation. 

In Japan, SDM research has not undergone considerable advancements. As the measurement scale is now available in Japanese, SDM studies in Japan can be conducted using this common tool. This will help advance SDM research in Japan as findings obtained in Japan can now be compared with international findings.

The limitations of this study included the fact that the survey involved outpatients during their initial consultation at primary care facilities only in specific regions of Japan. Thus, it is difficult to generalize the results of this study to the entire Japanese population. Simultaneously, efforts should be made to improve the processes and outcomes of decision-making support from the patient’s perspective. Furthermore, the sample size was at the lower bound of what is necessary to perform confirmatory factor analysis via maximum-likelihood estimation ^(27)^.

In this study, we did not include certain factors such as the socioeconomic status (e.g., income), primary diagnosis of the patients, comorbidities of the patients, or family members. These are expected to be important, but their relationship with SDM could not be examined and must be a future research theme. Moreover, the questionnaires for the patients in this study were completed anonymously, making it impossible to link them to medical data. For this reason, accurate data on the disease could not be obtained. The characteristics of SDM for each disease will be examined in further study. Taken together, the Japanese version of the SDM-Q-9 was found to be reliable and valid for use in Japanese primary care settings. The patient-perceived SDM experience was strongly associated with decisional conflict, and a component of it was suggested to be associated with patient age and marital status.

## Article Information

### Conflicts of Interest

None

### Sources of Funding

This work was partially supported by the Geriatrics and Gerontology Research & Development Fund 29-5.

### Acknowledgement

The authors would like to express their grateful appreciation to all the staff members of the Tokyo-Hokuto Health Co-operative Association, Kawasaki Health Co-operative Association, Family clinic Hiratsuka, Kita-Tama Chuo Health Co-operative Association, the Hokkaido Centre for Family Medicine, and Professor Naoko Arimori.

### Author Contributions

Study concept, design, subject recruitment, data analysis, interpretation of data and preparation of manuscript: Dr. YG Study concept and preparation of manuscript: Dr. HM Study concept, design, subject recruitment and interpretation of data: Dr. DS Preparation of Manuscript: Dr. HA Interpretation of data and preparation of manuscript: Dr. LK Preparation of manuscript: Dr. IS Preparation of manuscript: Dr. MH Subject recruitment: Dr. KS Subject recruitment: Dr. TK

All authors have contributed to data collection and interpretation and critically reviewed the manuscript.

All authors read and approved the final manuscript.

### Approval by Institutional Review Board (IRB)

Approval code: 913

Name of institution: National Center for Geriatrics and Gerontology in Japan, Obu, Aichi, Japan.

Date of approval: 28 March 2016
